# Characterization of tumor infiltrating lymphocytes in paired primary and metastatic renal cell carcinoma specimens

**DOI:** 10.18632/oncotarget.4572

**Published:** 2015-07-10

**Authors:** Marina K. Baine, Gabriela Turcu, Christopher R. Zito, Adebowale J. Adeniran, Robert L. Camp, Lieping Chen, Harriet M. Kluger, Lucia B. Jilaveanu

**Affiliations:** ^1^ Department of Medicine, Yale University School of Medicine, New Haven, CT, USA; ^2^ Department of Dermatology, Carol Davila University of Medicine and Pharmacy, Bucharest, Romania; ^3^ Department of Biology, School of Health and Natural Sciences, University of Saint Joseph, West Hartford, CT, USA; ^4^ Department of Pathology, Yale University School of Medicine, New Haven, CT, USA; ^5^ Department of Immunobiology, Yale University School of Medicine, New Haven, CT, USA

**Keywords:** tumor infiltrating lymphocytes (TILs), renal cell carcinoma (RCC), primary, metastatic

## Abstract

Renal cell carcinoma (RCC) is one of the most chemo- and radio-resistant malignancies, with poor associated patient survival if the disease metastasizes. With recent advances in immunotherapy, particularly with PD-1/PD-L1 blockade, outcomes are improving, but a substantial subset of patients does not respond to the new agents. Identifying such patients and improving the therapeutic ratio has been a challenge, although much effort has been made to study PD-1/PD-L1 status in pre-treatment tumor. However, tumor infiltrating lymphocyte (TIL) content might also be predictive of response, and our goal was to characterize TIL content and PD-L1 expression in RCC tumors from various anatomic sites. Utilizing a quantitative immunofluorescence technique, TIL subsets were examined in matched primary and metastatic specimens. In metastatic specimens, we found an association between low CD8+ to Foxp3+ T-cell ratios and high levels of PD-L1. High PD-L1-expressing metastases were also found to be associated with tumors that were high in both CD4+ and Foxp3+ T-cell content. Taken together these results provide the basis for combining agents that target the PD-1/PD-L1 pathway with agonist of immune activation, particularly in treating RCC metastases with unfavorable tumor characteristics and microenvironment. In addition, CD8+ TIL density and CD8:Foxp3 T-cell ratio were higher in primary than metastatic specimens, supporting the need to assess distant sites for predictive biomarkers when treating disseminated disease.

## INTRODUCTION

Renal cell carcinoma (RCC) is one of the more common solid malignancies, with over 61,000 new cases diagnosed each year in the United States alone. It is responsible for almost 14,000 annual deaths [1], and despite the evolution of new therapies in the last several decades, remains difficult to treat when diagnosed in advanced stages. Due to its notoriously chemoresistant phenotype, for decades the mainstay of therapy for advanced RCC had been systemic immunotherapy, namely IL-2 and IFN-α. Development and FDA approval of VEGF- and mTOR-targeted agents in the early 2000s resulted in improved outcome in patients with metastatic RCC, but the 5-year-survival rate has remained less than 15% [2].

In the last decade, in the context of growing scientific understanding of the interplay between cancer and the immune system, new agents targeting immune check-point molecules have been developed and tested in advanced solid tumors, including RCC. Monoclonal antibodies targeting T-cell surface receptors responsible for downregulation of immune response to tumor antigens, such as PD-1 and CTLA-4, have been designed and demonstrated impressive improvements in solid tumor responses and patient survival in several clinical trials [3, 4], including a recently completed phase III trial (NCT01668784). A phase I trial combining inhibitors of PD-1 and CTLA-4 demonstrated further increase in response rates that were ongoing in 80% of responders at the time of study publication [5]. This combination is now being further studied in several randomized trials (NCT02210117, NCT01472081, and NCT02231749). Similar outcomes have been reported with a monoclonal antibody targeting one of the PD-1 ligands (PD-L1) that is expressed on antigen-presenting cells and on tumors [6]. An alternative therapeutic approach to blocking immunosuppressive checkpoint molecules has been to enhance T-cell activation and anti-tumor response via agonists to co-stimulatory surface receptors, such as 4-1BB, GITR, CD27, and OX40. Studies of agents targeting OX40 and OX40-ligand are in early pre-clinical and clinical phases, but have shown significant success in animal models [7–9]. Similarly, there are early phase clinical trials of CD27, GITR and 4-1BB targeted agonists that are recruiting patients with advanced solid malignancies including RCC (NCT01460134, NCT01239134, NCT01307267, NCT01471210).

In the setting of these successes, characterizing the tumor immune microenvironment has become ever more essential for deciphering the mechanisms of tumor immune evasion and determining how to harness the immune system in fighting cancer. One approach to this has been to examine the association between tumor infiltrating lymphocyte (TIL) content with clinicopathologic characteristics of the tumors and patient outcome. It has been demonstrated, for instance, that in RCC high density CD4+ T-cell infiltrate is associated with unfavorable tumor characteristics and poor prognosis [10, 11]. There is also a preponderance of evidence suggesting an association of high Foxp3+ regulatory T-cell (Treg) content among both peripheral blood mononucleated cells (PBMCs) and TILs with metastatic relapse and poor prognosis [12–15]. Furthermore, Tregs isolated from TILs demonstrated more immunosuppressive activity than those from PBMCs [16]. Results from a study by Siddiqui et al, however, suggested that the negative effect on survival is attributed to Foxp3- rather than Foxp3+ Tregs [17]. The role of cytotoxic CD8+ TILs has been controversial in the context of RCC, with several studies demonstrating conflicting results [10, 18–22].

Another approach to examining the complex tumor microenvironment has been to look at the relative ratios of the various TIL subsets, as a measure of balance between the active anti-tumor lymphocyte population and immunoregulatory/suppressive population. High ratios of CD8+ to Foxp3+ T-cells among TILs, for example, have been shown in a variety of solid tumors to be associated with favorable tumor characteristics, response to therapy and radiation, and improved patient survival [23–26]. In an extensive meta-analysis study looking at the prognostic value of TILs in all cancer types, Gooden *et al*. concluded that CD8+ and Foxp3+ TILs ought to be examined together when establishing prognostic relevance, as the ratio of the two T-cell subtypes was most strongly associated with survival, with a hazard ratio (HR) of 0.48 [27]. To our knowledge, since the publication of the meta-analysis there have been no reports examining the CD8:Foxp3+ T-cell ratio in the setting of renal cell carcinoma, and differences in T-cell content in primary versus metastatic specimens are unknown. This distinction is critical given that in some patients the bulk of the unresectable disease is in the kidney while in others, distant metastases might be the primary target of systemic therapy. In the present study we compared TIL content in paired primary and metastatic tumors from RCC patients with stage IV disease and examined the associations with clinicopathologic tumor characteristics and corresponding levels of tumor PD-L1 expression.

## RESULTS

### Immunofluorescence for TIL content

To characterize TIL subpopulations in primary and metastatic RCC, the aforementioned TMA containing 4 primary and 4 matched metastatic cores, was stained with antibodies to CD3+, CD4+, CD8+ and Foxp3+ TIL subsets. The latter demonstrated exclusively nuclear staining, confined to T-cells, as indicated by co-localization with the CD3+ signal. No Foxp3 positivity was observed in tumor tissue. CD3, CD8, and CD4 signals were membranous. Tumor cores were excluded from analysis if tissue was lost during staining or exhausted from the block, if background was too intense to accurately isolate from the true signal, or if there was extensive necrosis (>90% of histospot). Necrotic regions were not included in the analysis.

The greatest percent area per histospot (d = 0.6 mm, A = 0.28 mm^2^) of CD3+ TILs ranged from 0.02% to 66%, with a median of 4.4%. For CD4+ TILs the range was 0–38%, with a median of 1.7%. For CD8+ and Foxp3+ lymphocytes, the percent area ranges were 0–33% and 0–1.5%, with medians of 1.8% and 0.05%, respectively. For CD3+, CD4+, and CD8+ T-cells, each 1% of the area corresponded to approximately 75 cells, while for Foxp3+ TILs, 1% was equivalent to 167 cells.

### Associations of TIL subsets within and between primary and metastatic specimens

With the exception of CD3, the percentage of fluorescent area values for all TIL subsets were correlated between the two TMAs, with a Pearson correlation coefficient *r* > 0.6. Densities of CD3+ TILs were found to be more variable (*r* = 0.41), particularly in primary RCC tissue (*r* = 0.28), where this subset was most heterogeneous.

To determine whether TIL content in metastatic disease can be predicted from primary nephrectomy tissue and vice versa, we assessed the correlation between the densities of each TIL subset (defined by maximum percent area of fluorescence per histospot) in matched primary and metastatic lesions by linear Pearson correlation test. Weak associations were found between CD4+ and CD3+ T-cell density in matched primary and metastatic tumors, with *r* = 0.3 and 0.4, respectively. This association was stronger for the CD8+ and Foxp3+ TIL subsets (*r* = 0.5 and 0.6, respectively).

**Figure 1:**
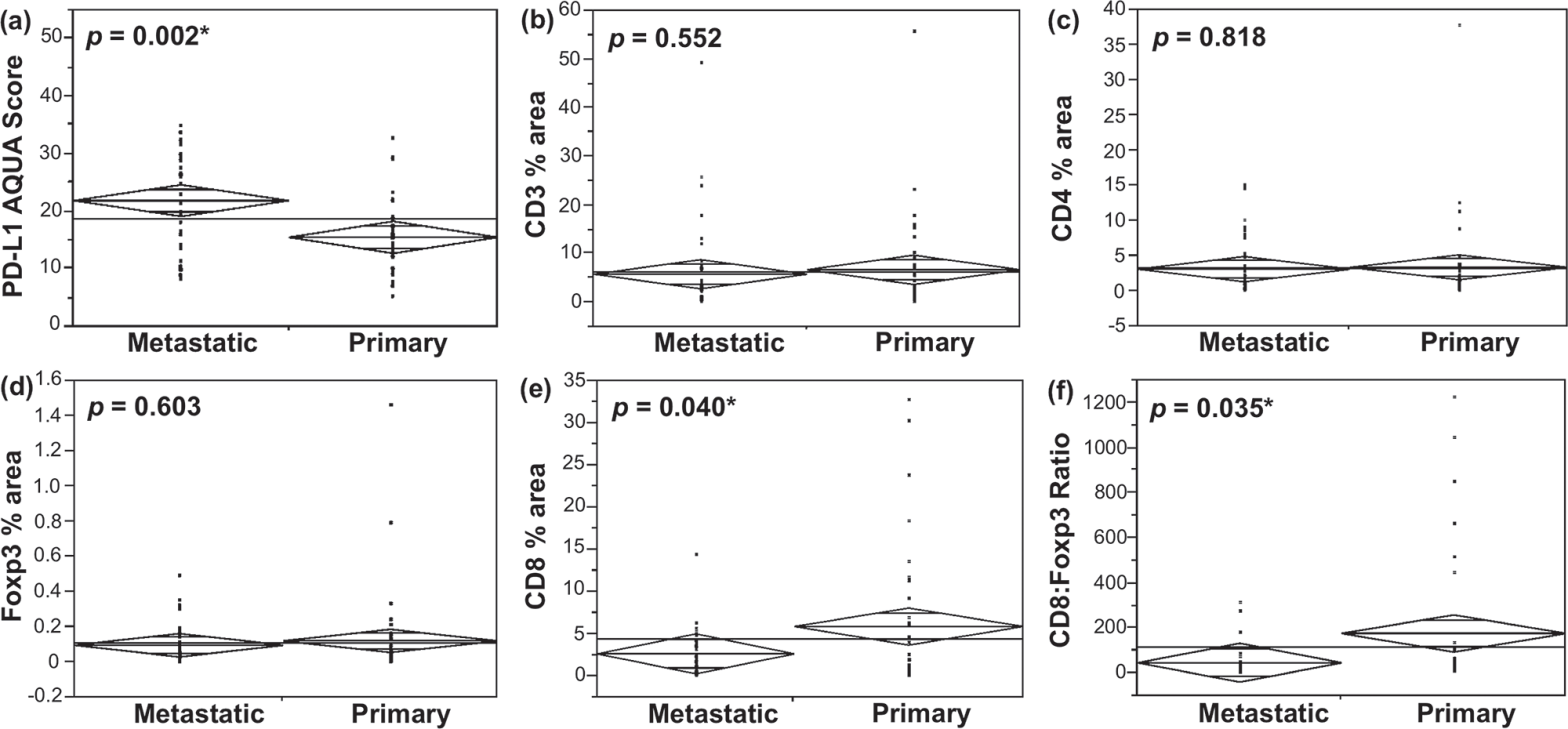
Tumor PD-L1 and TIL subtype distribution between primary and metastatic renal cell carcinoma **a.** One-way analysis of variance (ANOVA) of PD-L1 expression (continuous intensity scores) in RCC lesions demonstrated significantly higher levels of PD-L1 in metastases than in primary tumors [28]. **b–d.** Densities of CD3+, CD4+ and Foxp3+ TILs (% area per histospot), respectively, did not differ between metastatic and primary lesions. **e.** Primary RCC tumors had higher CD8+ TIL content than metastases, with the ratio of CD8+ to Foxp3+ T-cells demonstrating the same pattern **f.**

We have previously shown higher levels of PD-L1 expression in metastatic RCC lesions compared with matched primary tissue [28] (also shown in Figure 1). To probe for any such differences in the tumor microenvironment, we compared the TIL density for each subset between primary and metastatic specimens. Only CD8+ T-cell density was significantly different between primary and metastatic tissue, with higher density in primary RCC lesions (*p* = 0.04), as shown in Figure 1. No such difference was observed for CD3+ and CD4+ TILs.

Although never examined in the context of RCC, the ratio of cytotoxic CD8+ T-cells to Foxp3+ T-cells has been evaluated in other tumor types in the context of clinical outcome [23–26]. In our patient cohort, although Foxp3+ TIL density did not differ between primary and metastatic lesions, CD8:Foxp3 T-cell ratio was higher in primary than in metastatic tumor tissue (*p* = 0.035, Figure 1).

**Figure 2:**
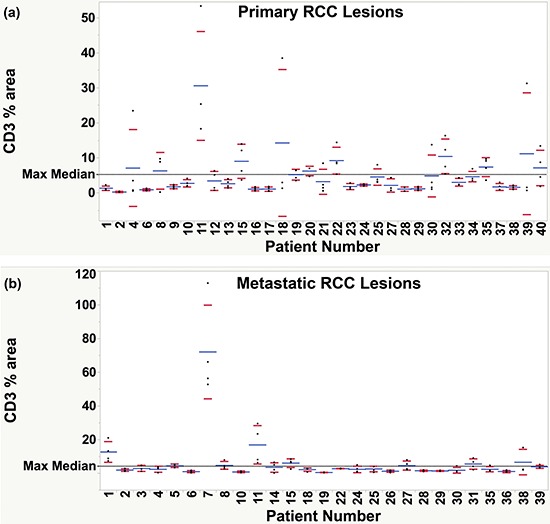
Examination of heterogeneity of the total T-cell infiltrate within primary and metastatic RCC lesions Percent fluorescence area of CD3+ signal was examined for each patient with at least three representative tissue cores. Each dot represents a separate histospot for the corresponding patient, numbered sequentially 1–40. Average % area of CD3+ T-cells is shown for each patient as a blue line. Red marks represent the lower and upper limits of the standard deviation. A reference line (black, dashed) was drawn at the selected threshold for defining high and low density infiltrate (median of the maximum CD3+ T-cell density for all patients). Greater variability in CD3+ T-cell content was observed among the primary specimens **a.** collected from each patient than among the metastatic cores **b.** as demonstrated by a greater proportion of patients with values spanning across the selected threshold.

Given that RCC tumors are known to be very heterogeneous, core needle biopsies might not accurately reflect the entire tumors. Many metastatic patients can undergo biopsy from either a primary or a metastatic site to determine diagnosis and likelihood of responding to therapies based on tumor microenvironment. To determine whether the primary or metastatic sites differed in overall T-cell content, we compared the distribution of CD3+ T-cell densities in primary and metastatic lesions in the different TMA histospots, which are of similar diameter to biopsies. As shown in Figure 2, the primary lesions tend to have a broader distribution of TIL content in different histospots than metastatic tumors, suggesting that if TIL content is used to predict response to therapy, biopsies from metastatic sites might better represent the entire tumor than biopsies from primary sites.

### Correlation between TIL subsets and patient and tumor characteristics

There was no significant difference between the densities of the examined TIL subsets in patients of different genders and ages (data not shown). It was noted, however, that the average AQUA score for PD-L1 expression was higher in metastatic lesions of patients who were younger than 50 years old (*p* = 0.047, data not shown).

There was no correlation between nuclear grade or disease stage at the time of nephrectomy and densities of CD3+, CD4+, CD8+, or Foxp3+ T-cell infiltrates (Table 1). Of note, all tumors larger than 10 cm were associated with a low % area of CD3+ and CD8+ TILs, defined as less than the median of the maximum total T-cell infiltrate identified by CD3-positivity (Table 1). No statistical difference was observed for any of the TILs when the size threshold for defining large tumors was set at 7 cm (data not shown).

**Table 1:** Correlation of TIL subsets and tumor PD-L1 with pathologic tumor characteristics

Marker	Nuclear Grade	Tumor size	Stage at nephrectomy
	Low = 1–2 High = 3–4	Large ≥ 10 cm	Early = 1–3 Advanced = 4
**CD3+**	χ^2^ = 0.45 *p* = 0.502	**χ^2^ = 7.18** ***p* = 0.007**	χ^2^ = 0 *p* = 1
**CD4+**	χ^2^ = 3.29 *p* = 0.070	χ^2^ = 1.79 *p* = 0.181	χ^2^ = 0.06 *p* = 0.805
**CD8+**	χ^2^ = 0.83 *p* = 0.362	**χ^2^ = 4.61** ***p* = 0.032**	χ^2^ = 3.06 *p* = 0.081
**Foxp3+**	χ^2^ = 0.16 *p* = 0.688	χ^2^ = 0.56 *p* = 0.454	χ^2^ = 1.04 *p* = 0.308
**CD8:Foxp3 Ratio**	χ^2^ = 0.001 *p* = 0.979	χ^2^ = 0.095 *p* = 0.758	χ^2^ = 0.33 *p* = 0.564
**Tumor PD-L1**	χ^2^ = 1.04 *p* = 0.307	χ^2^ = 1.22 *p* = 0.268	χ^2^ = 0.52 *p* = 0.473

It has previously been shown that RCC pulmonary metastases tend to respond better to immunotherapy and are associated with better prognosis than other sites [29–31]. To determine whether these clinical findings could potentially be attributed to the tumor microenvironment, we examined the TIL subset distribution in the lung and other metastatic sites (Table 2). For all sites, total T-cell infiltrate, as defined by CD3-positivity, was evenly distributed, such that there was no prevalence of high density of CD3+ TILs in any one particular organ (*p* = 0.730). The same was true for tumor PD-L1 expression (*p* = 0.463). There was no difference in the CD4+ and CD8+ T-cell content between the various anatomic sites. Notably, high density of Foxp3+ T-cell infiltrate was more likely to be found in lung than in other metastatic sites (*p* = 0.049).

**Table 2:** Distribution of TIL subsets at different sites of metastasis

Site of Metastasis	CD3+		CD4+		CD8+		Foxp3+		CD8:Foxp3 Ratio		Tumor PD-L1	
	LOW	HIGH	LOW	HIGH	LOW	HIGH	LOW	HIGH	LOW	HIGH	LOW	HIGH
**Lung**	7 (47%)	8 (53%)	10 (67%)	5 (33%)	10 (77%)	3 (23%)	4 (27%)	11 (73%)	6 (46%)	7 (54%)	9 (60%)	6 (40%)
**Other**	10 (53%)	9 (47%)	17 (81%)	4 (19%)	14 (82%)	3 (18%)	13 (59%)	9 (41%)	12 (71%)	5 (29%)	8 (47%)	9 (53%)
***P*-value**	0.730		0.332		0.713		0.049*		0.175		0.463	

### Association between Foxp3+ T-cells and tumor PD-L1 expression

**Figure 3:**
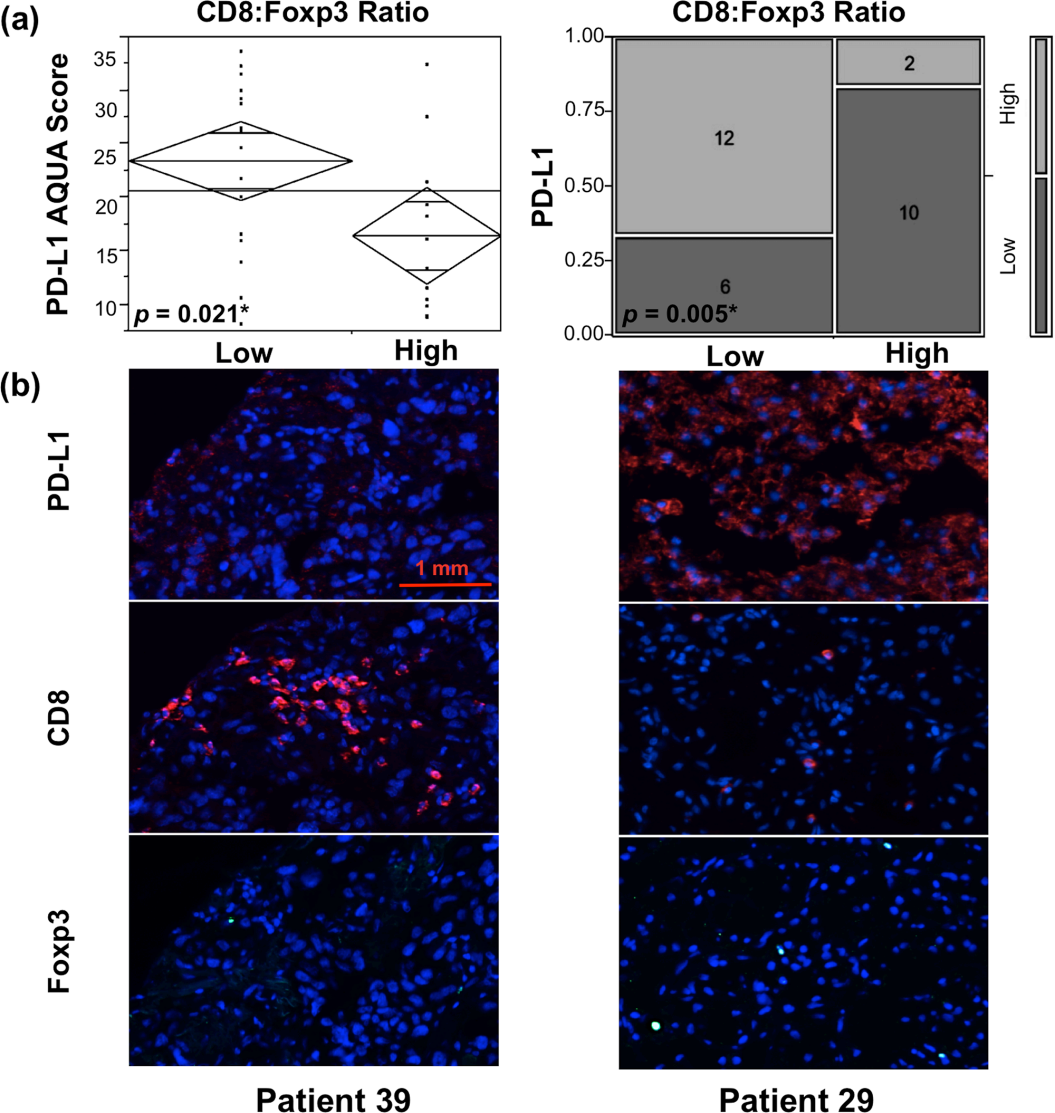
TILs with low CD8:Foxp3 T-cell ratio are associated with higher PD-L1-expressing metastatic RCC lesions Median values of PD-L1 AQUA scores and CD8+ to Foxp3+ TIL percent fluorescent area ratios were used as thresholds for defining low and high content of each. **a.** ANOVA and Chi square test of PD-L1 expression in RCC metastases, demonstrated higher tumor PD-L1 levels associated with low ratio of CD8+ to Foxp3+ T-cells. **b.** Representative 4 × 2.5 mm immunofluorescence images of metastases from patients 39 and 29 are shown. DAPI was utilized to visualize nuclei (blue), Cy5 to visualize PD-L1 and CD8 (red), and Cy3 to visualize Foxp3 (green). Tumor from patient 39 showed low PD-L1 expression and an associated T-cell infiltrate with high CD8+ and low Foxp3+ T-cell density, and a corresponding high CD8:Foxp3 ratio. Conversely, high PD-L1 expressing metastatic lesion from patient 29 was associated with a low CD8+ to Foxp3+ T-cell ratio.

PD-L1 expression by tumors has been shown to suppress T-cell function and induce a local immune tolerance, by mechanisms that are currently well-characterized [32, 33]. It has also been associated with poor prognosis and survival in many solid tumors, including RCC [18, 34–36]. Utilizing the same patient cohort as for the current study, our group has previously shown PD-L1 predominance in metastatic lesions [28]. In this context, we set out to determine the immune microenvironment of tumors associated with differential PD-L1 expression in primary and metastatic RCC. One of our most striking observations was that the CD8:Foxp3 T-cell ratio was inversely correlated with PD-L1 in metastatic lesions (Figure 3), such that higher tumor PD-L1 expression was associated with a low CD8:Foxp3 ratio (*p* = 0.021 by *t*-test, and *p* = 0.005 by Chi square test). Consistent with this result, the inverse ratio of Foxp3:CD8 T-cells was directly and significantly correlated with the level of PD-L1 expression by RCC metastases (data not shown). No such effects were observed in primary tissue, suggesting a different mechanism of immune evasion and tumor microenvironment regulation in primary and metastatic lesions. Neither Foxp3+ nor CD8+ TIL density alone had any correlation with tumor PD-L1 expression.

The density of total T-cell infiltrate, represented by CD3 positivity, did not correlate with tumor PD-L1 expression. Conversely, high Treg content, denoted by the ratio of Foxp3:CD3+ T-cells was significantly associated with higher PD-L1 expression in metastatic lesions (*p* = 0.049, data not shown). This relationship did not hold for the Foxp3:CD4 ratio, likely due to the presence of non-CD4+ Tregs. In the recent past, for instance, several studies have shown an important role of CD8+Foxp3+ Tregs in tumor immune evasion [37, 38].

**Figure 4:**
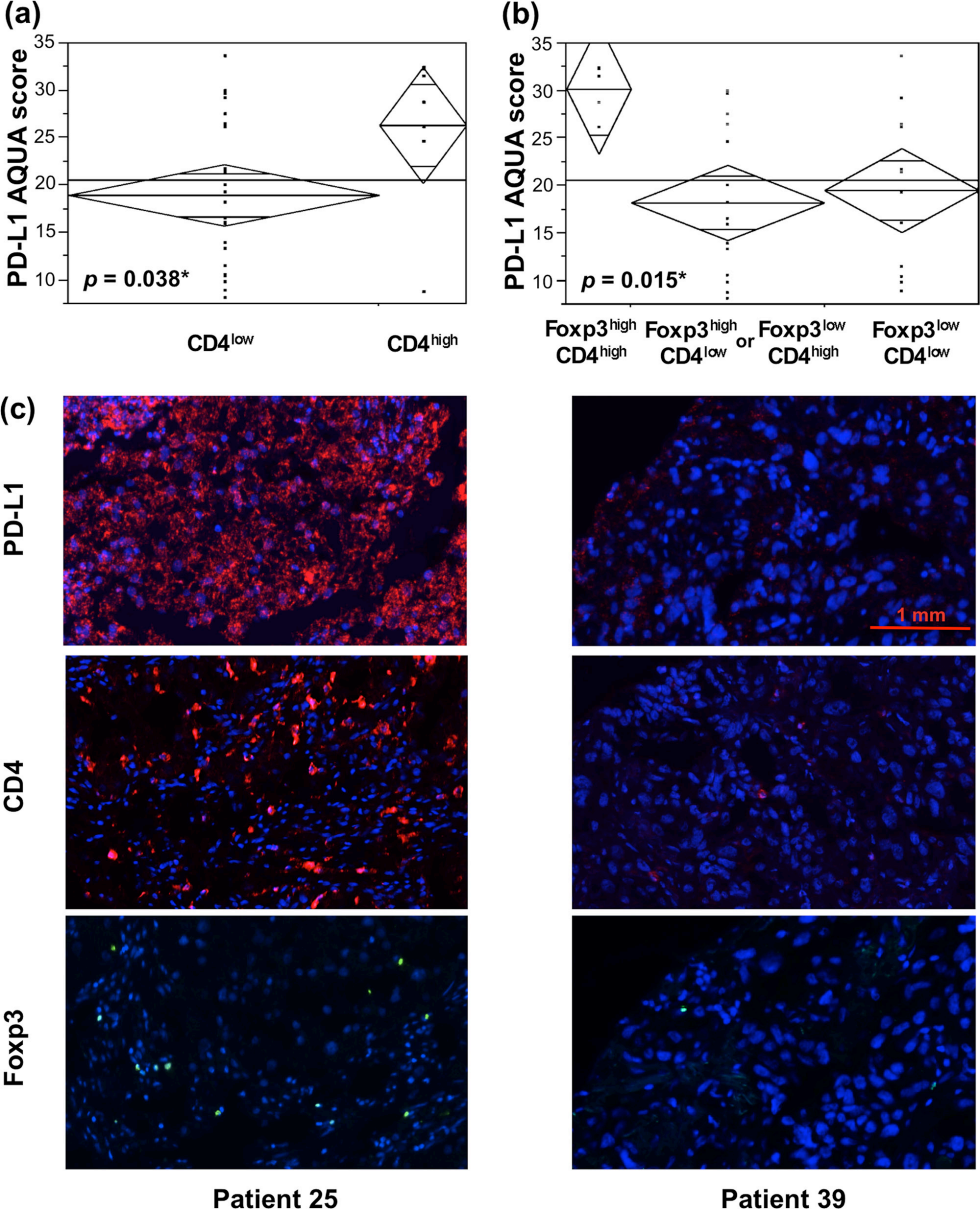
TILs with high CD4+ T-cell content and those that are both CD4- and Foxp3-high, are associated with higher PD-L1-expressing metastatic RCC lesions Median percent area of Foxp3+ T-cells was used as a threshold for high and low Foxp3+ content. For CD4+ T-cells, the cutoff for high density was the median area of the total T-cell infiltrate (CD3+ TILs). **a.** ANOVA of tumor PD-L1 expression in RCC metastases demonstrated higher PD-L1 levels associated with high CD4+ TIL content. **b.** Tumor PD-L1 expression was particularly high in metastases with a TIL population with high densities of both CD4+ and Foxp3+ T-cells (Foxp3^high^CD4^high^), as compared to Foxp3^low^CD4^low^, Foxp3^high^CD4^low^ or Foxp3^low^CD4^high^ subsets. **c.** Representative 4 × 2.5 mm immunofluorescence images of metastases from patients 25 and 39 are shown. DAPI was utilized to visualize nuclei (blue), Cy5 to visualize PD-L1 and CD4 (red), and Cy3 to visualize Foxp3 (yellow). Tumor from patient 25 showed high PD-L1 expression and an associated T-cell infiltrate with high CD4+ and Foxp3+ T-cell densities. Conversely, low PD-L1 expressing metastatic lesion from patient 39 was associated with low CD4+ and Foxp3+ T-cell contents.

CD4+ T-cell content among the TILs in RCC has been shown in several studies to correlate with unfavorable tumor characteristics and patient outcome [10, 11]. We observed a correlation between high CD4+ TIL density with high tumor PD-L1 expression in metastatic lesions (Figure 4, *p* = 0.038). As CD4+ T-cells comprise a heterogeneous group in that there are both tumor-reactive and suppressive populations, we set out to examine whether concomitant high Foxp3+ T-cell density contributed to its association with high-PD-L1-expressing metastases. We found that tumors that are high in both CD4+ and Foxp3+ T-cell content were associated with significantly higher average tumor PD-L1 expression (Figure 4, *p* = 0.014). CD4-high/Foxp3-low and CD4-low/Foxp3-high T-cell populations were grouped into one category because no significant difference was found between them and there were only two patients with the former T-cell composition. Nonetheless, significance was retained when the four aforementioned T-cell populations were considered separately (*p* = 0.039, data not shown).

## DISCUSSION

In the current study we assessed T-cell content in matched primary and metastatic samples from RCC patients and examined the association with PD-L1 expression and clinicopathologic covariates. Among the TIL subsets studied, only CD8+ and Foxp3+ TIL densities correlated best between primary and corresponding metastatic lesions. Although there was no significant correlation between TIL subset distributions and nuclear grade and stage at nephrectomy, it was noted that very large tumors (>10 cm) were associated with low CD3+ and CD8+ TIL density. This did not hold true when 7 cm was used as a size cut-off for defining large primary tumors. Although the number of cases included in this study was small, it appeared that lung metastases contained higher densities of Foxp3+ TILs than other sites, but no significant differences between the various metastatic sites were observed for other TIL subsets. Of note, we found an association between high CD8:Foxp3 T-cell ratio and lower PD-L1-expression in metastatic lesions. This relationship did not hold for the corresponding primary tumors. Furthermore, high PD-L1-expressing metastatic tumors, but not the corresponding primary lesions, were associated with TILs that contained high densities of both CD4+ and Foxp3+ T-cells.

In the era of rising precision medicine targeted immunotherapies have become the front-runners for treating a variety of solid malignancies. While several checkpoint inhibitors have already been FDA-approved for the treatment of metastatic melanoma, approval for use in patients with advanced RCC is pending. Activity of these agents in RCC is limited to subsets of patients, and there is therefore a great need to develop predictors of response to these therapies to facilitate patient selection. In developing candidate predictive biomarkers, a number of challenges unique to RCC arise since we may not know which tissue type (primary or metastatic) to test and how many biopsy cores are needed. Unlike other solid tumors in which asymptomatic primary lesions are not resected once the disease has metastasized, in RCC patients debulking nephrectomy is often done, even in the setting of metastatic disease. However, it is often the distant metastatic sites that are treated with the systemic therapy. Several RCC studies have demonstrated different immunohistochemical and mutational profiles in primary and metastatic lesions, while others have shown significant heterogeneity within large primary tumors [39–42]. This intra- and inter-tumor heterogeneity presents a major challenge to biomarker development. Our studies indicate that this challenge exists for precision medicine driven by both tumor-based molecular events and TIL characteristics. Moreover, future predictive biomarker studies and assay development will need to encompass both genomic aberrations and host immune response.

Consistent with RCC heterogeneity, we found that one may not utilize primary tumor tissue to predict the density of total T-cell infiltrate, as represented by CD3-positivity, in a corresponding metastatic lesion. This discordance can likely be attributed to the CD4+ helper T-cell content, as the remaining two subsets examined, CD8+ cytotoxic T-cells and Foxp3+ TILs, were concordant between primary and metastatic disease. Although CD8+ TILs are directly correlated in primary and metastatic lesions, the average density of this subset is significantly higher in primary tumors. Furthermore, the ratio of CD8+ to Foxp3+ T-cells, reflecting a balance between tumor-reactive and immunosuppressive TIL subpopulations, was also found to be higher in primary disease. This difference in immune microenvironment between primary and metastatic lesions suggests tumor adaptation as it progresses from early to advanced disease. Further molecular studies are required to determine the mechanism for this phenomenon.

The most common site of RCC metastasis is the lung, which is also reflected in our patient cohort. Pulmonary metastases tend to respond better to immunotherapy and are generally associated with better prognosis than extra-pulmonary sites [29–31]. As regulatory T-cells have been linked to treatment resistance [43] and poor survival [12–14], one would expect this TIL subset to predominate in extra-pulmonary metastases as compared to lung lesions. Nonetheless, in our study a greater proportion of lung metastases were associated with high densities of Foxp3+ T-cells, while the majority of other lesions had low Foxp3+ infiltrate. One possible explanation to this phenomenon is a relative scarcity of tumor-reactive T-cells in extra-pulmonary lesions as compared to lung, as would be represented by a low CD8:Foxp3 ratio. Although there was a trend toward such a pattern (*p* = 0.175), due to insufficient sample size, this ratio could not be used to accurately account for the clinical distinction between the different sites of metastasis. Alternatively, it is possible that patients who underwent resection of lung lesions and had available tissue for these studies were those with massive lung lesions requiring debulking surgery and not the lower risk patients. Larger studies examining relative distributions of cytotoxic T-cells and Tregs among different sites of metastases are needed to decipher their effects on organ-specific therapeutic response.

Unlike the effect of Foxp3+ Tregs, the role of cytotoxic CD8+ TILs in the setting of RCC has been controversial, with several studies demonstrating conflicting results [10, 18–22]. This could be attributed to technical factors, including antibodies used to label CD8+ T-cells, techniques used to detect them (immunohistochemistry versus flow cytometry), and tumor site being examined (peritumoral versus intratumoral TILs). Potentially, of most relevance was the ability of the studies to differentiate between the effector cytotoxic CD8+ T-cells and their exhausted counterparts, expressing high levels of immune checkpoint molecules in a dysfunctional immune environment. When dichotomized in such a way, Giraldo et al was able to clearly demonstrate good prognosis with the former CD8+ T-cell population, and poor prognosis with the latter [19]. Similarly, Nakano et al showed that TILs with high CD8+ T-cell content that exhibited high proliferative activity were associated with improved survival among patients with advanced RCC [21]. Although we were unable to distinguish between these two T-cell subpopulations, our approach to assessing the contribution of CD8+ T-cells was to examine their relative ratio to the suppressive Foxp3+ T-cells, which has been shown to be an effective surrogate for a physiologic threshold of anti-tumor activity [27].

In our study we found an association between high CD8:Foxp3 ratio and low PD-L1-expressing metastatic lesions and vice versa (*p* = 0.005). Furthermore, we found that metastatic tumors with higher surface PD-L1 expression were associated with a CD4-high/Foxp3-high TIL subpopulation (*p* = 0.015), which would denote an unfavorable immune microenvironment in untreated patients. However, precisely these patients might be more likely to respond to targeted immunotherapies. Notably, this relationship did not hold for primary RCC tumors, which underscores the need to conduct predictive biomarker studies on the tumor site that is being treated.

One possible approach to treating patients with high tumor PD-L1 expression and associated low CD8:Foxp3 T-cell ratios is the addition of immune-stimulatory molecules, such as agonists of CD27, GITR, 4-1BB, OX40 and others. These molecules, belonging to the tumor necrosis factor receptor (TNFR) superfamily that signal through NF-κB, Jun, and p38 to induce inflammation, have been assessed for targeted immunotherapy in early phase clinical trials (NCT01460134, NCT01239134, NCT01471210, NCT01307267, NCT01644968). Although there is functional overlap among these receptors, each one has a predominant role in immune regulation. Ligation of CD27, for example, primarily results in development of T-cell memory [44], which could potentially lead to long-term tumor control. While CD27 is rarely found on the surface of regulatory T-cells, GITR was first discovered in these cells as a crucial player in direct suppression of their activity resulting in loss of immune tolerance [45]. Although the role of 4-1BB in regulation of Treg activity has proven to be complex, ligation of this receptor has been shown to lead to potent stimulation of natural killer (NK) cells and preferential support of activated CD8+ T-cell proliferation, differentiation and survival [46, 47]. Finally, OX-40 receptor ligation has been shown to simultaneously support proliferation and effector function of CD4+ and CD8+ T-cells and inhibit IL-10 production and suppressive function of Tregs [48, 49]. On the basis of our results, it would therefore follow, that targeting the tumor PD-L1 receptor in combination with stimulation of the OX-40 receptor on T-cells might be an effective strategy in treating RCC metastases. Although targeting a combination of the other TNFR members may achieve a similar goal, exploiting the downstream effects of OX-40 signaling could potentially achieve a favorable tumor microenvironment rich in effector cytotoxic CD8+ T-cells and depleted in Tregs.

In summary, our results demonstrate a predominance of Foxp3+ T-cells in lung metastases compared to other organs, but larger studies are needed to confirm this result. We have also shown that although CD8+ and Foxp3+ T-cells are concordant between primary and metastatic lesions, primary RCC is associated with a higher CD8+ TIL density and CD8:Foxp3 T-cell ratio, supporting the need to assess metastatic sites when treating distant disease. Of further relevance was our finding that there is a significant correlation between tumor PD-L1 expression and TIL content in metastatic RCC lesions. Higher levels of tumor PD-L1, were associated with low relative ratios of CD8+ to Foxp3+ T-cells. As there are effective immune therapies that target both of these aspects of RCC metastases, our work further supports combining antagonists to immune inhibition, directed against the PD-1/PD-L1 interaction, with agonists of immune expansion and proliferation. Prospective studies of PD-L1 and TIL content in patients treated with these drugs will likely improve the therapeutic ratio.

## MATERIALS AND METHODS

### Tissue microarray (TMA) construction

TMAs were constructed from a cohort of forty patients who had undergone both nephrectomies and metastatectomies between 1978 and 2011. Specimen accrual and TMA construction was approved by the Yale University Institutional Review Board. For each patient, primary and metastatic sites were represented with four 0.6 mm cores taken from different areas of each specimen, for a total of eight cores per patient. To determine reproducibility of staining, the tissue was embedded into two separate TMA blocks, each containing two matched primary and metastatic cores (four cores per patient on each TMA block). For normalization between the two blocks, several identical cell lines, cored from paraffin-embedded cell pellets, were added to each TMA block. Collection of specimens, clinical information, and tumor characteristics, complete with size, nuclear grade, and histology, were previously described [42, 50]. The cohort included 25 male and 15 female patients, ranging in ages from 17 to 72 years old, with median age at diagnosis of 56. The majority of the patients (37 of 40, 93%) had pure clear cell renal cell carcinoma (ccRCC), and only three had mixed sarcomatoid and clear cell histology (7%). Patients were treated with a range of anti-tumor agents, with only two of thirty-four receiving VEGF or VEGFR inhibitors. None of the patients had been exposed to targeted immunotherapies. The most represented metastatic site was the lung (15 patients), followed by bone (7), lymph nodes, skin, soft tissue, liver, adrenal glands, colon, and pituitary gland. To verify antibody specificity, we used normal lymph node tissue.

### Immunofluorescent staining of tumor-infiltrating lymphocytes (TILs)

For T-cell detection, serial TMA cuts were deparaffinized by heating at 60°C for 30 minutes, followed by three 10-min xylenes incubations. Tissue was rehydrated by serial emersions in 100% EtOH, twice for 3 minutes each, and once for 5 minutes in deionized H_2_O. Antigen retrieval was achieved by pressure-cooking the slides for 15 minutes in Tris-EDTA buffer (100x, cat# sc-296654A, Santa Cruz) brought to pH 9 using a 1M NaOH solution. Endogenous peroxidases were blocked by incubation for 30 minutes in a 0.75% H_2_O_2_ solution in methanol. Slides were washed twice for 30 seconds in tap water and non-specific binding sites were blocked with 0.3% bovine serum albumin (BSA) in TBS for an additional 30 minutes. The slides were washed and incubated overnight at 4°C with a primary antibody mixture in 0.3% BSA/TBS.

CD8 surface T-cell marker (mouse anti-CD8 antibody, 1:800, cat# M7103, DAKO) and Foxp3 nuclear marker (mouse anti-Foxp3 mAb, clone 236A/E7, 1:200, cat# ab20034, Abcam) were co-stained with rabbit anti-CD3 antibody (1:1500, cat# A0452, DAKO). To visualize CD3+ T-cells, goat anti-rabbit HRP-conjugated polymer backbone (EnVision™, DAKO) was applied to tissue for 1 hour for signal amplification, followed by 10 minutes in Cyanine-3-tyramide. Slides were washed in TBST and HRP was quenched by incubating twice for 7 minutes with 100mM benzoylhydrazine (Acros Organics, New Jersey, USA) and 50 mM hydrogen peroxide solution in PBS. For Foxp3 and CD8 visualization, goat anti-mouse HRP-conjugated polymer backbone (EnVision™, DAKO) was applied to slides for 1 hour, followed by 10 minutes in Cyanine-5-tyramide. For detection of CD8+ T-cells, this step was done before blocking HRP signal, and followed by CD3 visualization as described above. After multiple subsequent washes in TBST, nuclear compartment was visualized using a DAPI stain (1:300, Life Technologies, Carlsbad, CA). Cover slips were mounted with ProLong^®^ Gold antifade reagent with DAPI (cat# P-26931, Life Technologies, Carlsbad, CA).

CD4+ T-cells were detected using mouse mAb to CD4 (1:400, cat# M7310, DAKO), amplified with anti-mouse EnVision reagent, and visualized with Cyanine-5-tyramide, as above. After blocking HRP, CD4 was counterstained for 2 hours with a mixture of rabbit anti-Cytokeratin (1:100, cat# Z0622, DAKO), anti-CA9 (1:2000, cat# NB100-417, Novus Biologicals), and streptavidin-peroxidase polymer (1:100, cat# S2438, Sigma) to highlight renal tumor cells. Again, the signal was amplified by using goat anti-rabbit EnVision reagent as previously mentioned, and tumor was finally visualized with Cyanine-2-tyramide. Nuclear compartment was stained with DAPI, and coverslips were mounted as described above.

### Automated image acquisition and QUantitative Analysis (AQUA)

Images were captured and analyzed using previously described methods and quantitative algorithms [51]. Monochromatic, high-resolution images were obtained for each TMA histospot. Tissue autofluorescence assessed using ultraviolet epifluorescence from DAPI-stained slides was used to construct a total tissue mask (which included both tumor and surrounding stroma). Tumor cell mask was visualized as described above, utilizing a cocktail of anti-CA9 and anti-cytokeratin antibodies with streptavidin-peroxidase polymer (bound to endogenous biotin). To assess tumor-infiltrating lymphocyte (TIL) content, percentage of each CD3+, CD4+, CD8+, or Foxp3+ T-cell area per histospot was determined based on area occupied by the corresponding signal within total tissue mask. Tumor PD-L1 expression levels were previously determined utilizing a 5H1 clone of the mouse monoclonal antibody to PD-L1 (generated by Dr. Lieping Chen, Yale University, New Haven, CT) and results were reported [28]. Briefly, PD-L1 signal was analyzed within the tumor cell mask, and expressed as the average signal intensity within the assayed component (AQUA score), on a scale from 0 to 255. Histospots were excluded from analysis if they contained < 5% tissue, were > 90% necrotic (based on nuclear and tissue morphology), or had abnormal staining patterns.

### Statistical analysis

JMP version 10.0 software (SAS Institute, Cary, NC) was used for statistical evaluation of data. CD3+, CD4+, and CD8+ TIL densities were binarized using the median total T-cell infiltrate (represented by CD3 positivity) as a cutoff for low and high categories. Foxp3+ TILs were dichotomized into low and high groups by median percent area of Foxp3+ T-cells. Associations between these categories and clinicopathologic parameters were determined by the Chi-square test. Continuous mean tumor PD-L1 AQUA scores, obtained from previous studies [28], were correlated with dichotomized densities of TIL subsets by one-way analysis of variance (ANOVA). Data reproducibility was assessed by Pearson linear correlation between replicate histospots.
